# Development and Characterization of Clean-Label Hybrid Deli Ham with Pulse Flours as Partial Meat Replacers

**DOI:** 10.3390/foods15193541

**Published:** 2026-10-04

**Authors:** Lisiane Carvalho, Soundary Seriacaroupin, Adriana Ferreira, Cláudia S. C. Marques-dos-Santos, Teresa J. S. Matos, Isabel Sousa, Anabela Raymundo

**Affiliations:** 1 LEAF—Linking Landscape, Environment, Agriculture and Food Research Center, Instituto Superior de Agronomia, Universidade de Lisboa, Tapada da Ajuda, 1349-017 Lisboa, Portugal; soundary.seriacaroupin@gmail.com (S.S.); matosteresa@isa.ulisboa.pt (T.J.S.M.); isabelsousa@isa.ulisboa.pt (I.S.); anabraymundo@isa.ulisboa.pt (A.R.); 2 R&D—Department, Primor Charcutaria-Prima, S.A., Gavião, 4760-003 Vila Nova de Famalicão, Portugal; adriana.ferreira@primor.pt; 3 CEF—Forest Research Centre, Associate Laboratory TERRA, Instituto Superior de Agronomia, Universidade de Lisboa, Tapada da Ajuda, 1349-017 Lisboa, Portugal; cms@isa.ulisboa.pt

**Keywords:** meat reduction, plant-based proteins, flexitarian diets, sustainability

## Abstract

Growing interest in sustainable and clean-label foods has increased the need for meat products with reduced animal meat content. This study comparatively characterized three deli ham formulations: a conventional control, a hybrid formulation in which 50% of the pork meat mass was replaced with chickpea flour, red lentil flour and potato protein isolate, and a clean-label hybrid formulation with the same meat replacement. For physicochemical, nutritional, texture and rheological analyses, one production batch was prepared for each formulation. A second, independently produced batch of each formulation was prepared for the sensory evaluation. The hybrid formulations showed lower moisture (48%) and in vitro protein digestibility (66%) than the control (78% and 87.4%, respectively) although all samples exhibited predominantly elastic behaviour. Mineral content and in vitro bioaccessibility varied according to the mineral and formulation. From a nitrogen footprint perspective, the hybrid formulations exhibited lower estimated nitrogen footprint values than the control under the assumptions and system boundaries applied in the model. Overall, sensory responses differed among formulations for several evaluated attributes, indicating differences in sensory acceptance across formulations. These findings show that partial replacement of pork meat modified the physicochemical, nutritional, technological and sensory characteristics of deli ham, while providing a potential approach for reducing the modelled nitrogen footprint of the product.

## 1. Introduction

The environmental implications of meat production, including greenhouse gas emissions, biodiversity reduction, and intensive land use, have increased interest in strategies that reduce the proportion of animal-derived ingredients in food products. Substituting meat proteins with alternative plant-based proteins might influence the environmental impact and human health in a positive way [1,2,3]. In parallel, the industry has experienced a shift towards less refined and more natural food solutions, leading to the emergence of the clean-label trend [4]. Simultaneously, the global market for meat analogue products has expanded rapidly, driven by increasing consumer demand for convenient, protein-rich foods, with sensory attributes similar to meat. Market projections indicate that the global meat alternatives market is expected to reach approximately USD 8.1 billion by 2026, growing at a compound annual growth rate (CAGR) of 7.8% between 2019 and 2026 [5].

Within this context, Europe represents the leading market, accounting for 51.5% of the global share, highlighting its dominant role in the development, commercialization, and consumption of meat alternative products. As a result, manufacturers face increasing pressure to identify natural functional ingredients, such as colourants, flavourings and preservatives, capable of fulfilling technological requirements while aligning with clean-label expectations [6]. In addition, studies have identified segments of consumers characterized by lowering meat consumption, an ongoing reduction, or an interest in reducing meat consumption [1,7,8].

Hybrid products, developed with animal and plant-based proteins, can provide an intermediate approach between conventional meat products and fully meat-free alternatives. By partially replacing meat with plant-derived ingredients, hybrid products may modify the nutritional composition and environmental burden of the final product while retaining some of the technological and sensory characteristics associated with meat products [9]. Several hybrid meat products have been launched and investigated in different markets, including sausages, burgers, meatballs, and minced meat, and may demonstrate higher sensory acceptance than their plant-based alternatives [10]. However, most available research has focused on these product categories, whereas comparatively less information is available on hybrid deli ham, a processed meat product in which the formation of a cohesive, sliceable, and stable matrix is particularly important.

Consumer demand for plant-based protein sources is increasing. Pulse flours (pea, chickpea, bean, lentil and lupin), with generally 13% to 43% protein content, are potential ingredients for incorporation into meat products, but they must be carefully selected to obtain a stable matrix that reduces water and fat losses while enhancing interactions with meat proteins [11,12]. These functional properties are mainly associated with the composition of pulse flours, as the presence of proteins together with starch and fibre promotes molecular interactions that facilitate gel formation during heating and cooling. Additionally, flour components help retain water and fat during the cooking process, thereby increasing the cooking yield [12].

Research on hybrid meat products highlights the need to investigate their structural stability in order to develop products with appropriate processing performance and quality characteristics. These studies have highlighted that during the manufacture of hybrid meat products, ingredient selection and meat-to-vegetable protein ratio, as well as process parameters (temperature, time, salt, sequence of addition of the ingredients, etc.), are crucial for optimizing the resulting hybrid meat products [10,13,14].

The development of innovative meat products to address convenience, affordability and well-being benefits is essential for the meat industry to attract consumers interested in novel alternatives, such as hybrid products. However, sensory evaluation studies on these products remain limited. Although some studies have shown that hybrid products can taste as good as, or even better than, their meat-only counterparts [10,15], further research is needed to better understand their sensory characteristics. Moreover, the flavour profile of products such as cooked hams can be optimized through the incorporation of natural flavourings and spices, allowing manufacturers to enhance consumer acceptance [16].

Previous work by the authors investigated the technological performance of a conventional control and a hybrid clean-label deli ham containing chickpea flour, red lentil flour and potato protein isolate, with 50% pork meat replacement and a 29% reduction in salt [17]. That study focused primarily on instrumental texture and rheological behaviour. In the present study, these two formulations were retained and an additional hybrid formulation, without the clean-label modifications, was included to provide a broader comparison among conventional and hybrid clean-label deli hams. In addition, a previous study by the authors investigated a fully meat-free clean-label deli ham alternative, addressing related aspects such as texture, rheology, colour, composition and sensory evaluation [6]. However, the fully meat-free formulation and product development approach differed from those of the formulations evaluated in the present study. The present study therefore extends the previous technological characterization by including an additional hybrid formulation and by evaluating broader nutritional, compositional, mineral bioaccessibility, antioxidant, environmental and consumer acceptance outcomes across the three formulations. Thus, while previous studies provide relevant technological and product development information, the present work provides a broader comparative characterization of conventional, hybrid and hybrid clean-label deli ham formulations.

The development of hybrid meat products, in which part of the pork is replaced by plant-based proteins such as pulse flours, has been investigated as a strategy to reduce the environmental impacts associated with conventional meat production. In particular, livestock production is a major contributor to reactive nitrogen (N) emissions, which are linked to several environmental issues. Given the growing concerns around the environmental impacts of reactive nitrogen (N), the N footprint concept was created by Leach et al. [18] to provide a quantitative metric framework for assessing nitrogen emissions linked to consumption and production systems. The amount of N released to the environment as a consequence of food production is an important anthropogenic reactive N source. Therefore, this calculation provides a transparent metric to identify hotspots of inefficiency, compare food products and diets, and support strategies aimed at reducing environmental impacts while maintaining nutritional adequacy [19,20].

Despite increasing interest in hybrid meat products, it remains unclear how partial replacement of pork meat with pulse flours and potato protein affects the combined nutritional, physicochemical, digestive, mineral bioaccessibility, antioxidant, environmental and sensory characteristics of deli ham, particularly when conventional and clean-label hybrid formulations are compared. The present study therefore aimed to comparatively characterize three deli ham formulations: a conventional control, a hybrid formulation containing chickpea flour, red lentil flour and potato protein isolate, with 50% pork meat replacement and a 29% reduction in salt, and a clean-label hybrid formulation with the same level of meat replacement.

## 2. Materials and Methods

### 2.1. Formulation and Preparation of the Hams

Three deli ham formulations were developed: (i) a control (76% pork meat as the protein source); (ii) a hybrid (non-clean-label) with 50% of the pork meat replaced by a blend of chickpea flour, red lentil flour, and isolated potato protein; and (iii) a clean-label hybrid with the same level of meat substitution but with no preservatives. The chemical composition (Table 1) of the pulse flours and pork meat used in the development of the hams was provided by the industry supplier.

The control formulation (i) included pork meat, water, ice, salt, sodium tripolyphosphate, carrageenan and nitrate. The hybrid formulation (ii) was prepared with the same base ingredients, with 50% of the pork meat mass replaced by a blend of chickpea flour, red lentil flour, and isolated potato protein, and additionally included oil, psyllium, natural colourant, and aroma. The aroma consisted of a ham flavouring mix, used to provide flavour without an additional technological function. The natural colourant was composed of sugars. The clean-label hybrid formulation (iii) differed from (ii) by replacing sodium tripolyphosphate with a commercially available natural alternative, described in the ingredient’s technical data sheet as a natural beef broth flavouring, and excluding carrageenan and nitrate. Both hybrid formulations (ii and iii) also presented a 29% reduction in salt content compared with the control. The detailed composition of the three formulations is presented in Table 2.

In this study, the term “clean-label” was operationally defined as a formulation approach focused on reducing or replacing selected conventional additives commonly associated with processed meat products. Accordingly, the clean-label oriented hybrid formulation was developed without nitrate and carrageenan and with sodium tripolyphosphate replaced by a natural source alternative. Functional ingredients required for product structure, processing performance, and sensory characteristics, including potato protein isolate, psyllium, natural colourant and aroma, were retained. Therefore, this terminology is used to describe the formulation strategy adopted in this study.

For ham preparation, the ingredients were mixed using a Thermomix TM31 (Bimby, Vorwerk, Wuppertal, Germany) following a specific order, with a gradual increase in speed from 3 to 7 over a total duration of 3 min. The mixture was then packed in synthetic casing and left to rest in the refrigerator at 4 °C for 12 h. After this period, the cooking process was performed in a water bath at 77 °C until the product’s core temperature reached 70 °C (approximately 50 min). After cooking and cooling, the products were placed back in the refrigerator at 4 °C for 3 days before being analyzed. Each batch consisted of a 350 g portion. For each formulation, a single production batch was prepared within a complementary study conducted in 2024 [17]. This same batch was used for the texture, rheological and physicochemical analyses reported in the present study. A second, independently manufactured batch of each formulation was prepared for the sensory evaluation.

### 2.2. Texture Profile Analysis (TPA)

The texture of the hybrid and control products was analyzed using a Texturometer TA-XT plus (Stable Micro Systems, Surrey, UK) [21]. Before the analysis, the samples were sliced into 10 mm slices using a SAS 120 C1 electric multi-purpose slicer (SilverCrest, TARGA GmbH, Soest, Germany). Texture Profile Analysis (TPA) was performed at 20 °C using a cylindrical probe (10 mm diameter) and a 30 kg load cell. Two consecutive compression cycles were applied to a penetration depth of 5 mm. The pre-test and test speeds were 1 mm/s, and the post-test speed was 3 mm/s. Five technical replicates from a single production batch were analyzed for each formulation. The measured parameters were firmness, determined as the peak force recorded during the first compression cycle; adhesiveness, calculated from the negative area of the force–time curve; and cohesiveness, calculated as the ratio of the area under the curve during the second compression cycle to that during the first compression cycle [22].

The TPA and rheological procedures and results for the control and clean-label hybrid formulations were previously reported in the 2024 conference proceeding [17]. These data are reproduced here as part of a broader comparative analysis that includes an additional hybrid formulation and more comprehensive analyses and interpretations of the product characteristics.

### 2.3. Rheology Measurement

Rheological measurements were performed using a controlled-stress rheometer Haake MARS III (Thermo Fisher Scientific, Waltham, MA, USA), equipped with a Peltier cooling system and an Eheim professional air compression system. Prior to the frequency sweep tests, a stress sweep was performed over a range of 0.1–100 Pa to determine the linear viscoelastic region (LVR). A stress of 10 Pa, within the LVR, was subsequently selected for the frequency sweep measurements. The mechanical spectra, G′ (storage modulus) and G″ (loss modulus) as a function of frequency, over the range of 0.01–100 Hz were obtained [23]. Measurements were performed at 20 °C. The samples were cut to a thickness of 2.5 mm. A serrated parallel plate system with a 20 mm diameter (PP20) and a 2.5 mm gap (previously optimized) was used. The samples were protected from drying with a lid. Three technical replicates from a single production batch were analyzed for each formulation. Statistical comparisons between formulations were based on the storage modulus (G′) at a single frequency of 1 Hz.

### 2.4. Colour Measurements

The colour measurements were performed using a Chroma Metre CR 400 Series Konica Minolta (Osaka, Japan) calibrated to white standards prior to measuring. The samples were sliced into 10 mm thickness and then the colour was measured. The data L*, a* and b* were analyzed, where L* indicates brightness (values increase from 0 to 100), a* indicates the degree of redness or greenness (+60, red; −60, green) and b* indicates the degree of yellowness or blueness (+60, yellowness; −60, blueness). The difference between the control and hybrid samples was determined by the total colour difference (ΔE) using Equation (1) [24]:

(1) $$\Delta E=\sqrt{(L_{i}^{*}-L_{0}^{*}{)}^{2}+(a_{i}^{*}-a_{0}^{*}{)}^{2}+(b_{i}^{*}-b_{0}^{*}{)}^{2}}$$

The letter i indicates the hybrid sample and 0 the control sample. When comparing the samples, the observer detects distinct colours if the ΔE value is higher than 5 [25].

### 2.5. Physicochemical Analysis

The physicochemical analysis of the control and hybrid hams was carried out using three technical replicates from a single production batch for each formulation. Water activity (a_W_) was determined at 25 °C using a water activity metre (LabMaster-aw Neo, Lachen, Switzerland). The pH measurement was performed using a pH metre BASIC 20+ (Crison Instruments, Barcelona, Spain) coupled to a 50 11 T pH electrode (HACH, Rheineck, Switzerland).

Water holding capacity (WHC) and oil holding capacity (OHC) were measured following the method described by Kassama et al. [26]. These analyses were performed on the uncooked formulations. Briefly, 10 g of the sample was mixed with 40 mL of distilled water or oil in a centrifuge tube. The samples were maintained in a water bath at 22 °C for 30 min and then centrifuged at 1200× *g* for 30 min. After centrifugation, the phases were separated, and the corresponding wet and dry weights were determined. The results were expressed as the percentage of water or oil retained relative to the dry weight of the sample according to Equation (2).

(2) $$WHC\;or\;OHC\;(\%)=\left[\left(wet\;weight\;\left(g\right)-dry\;weight\;\left(g\right)\right)/dry\;weight\;(g)\right]\times 100$$

The crude protein content of the samples was estimated from the total nitrogen content determined using the DUMAS method (Thermo Quest NA 2100 Nitrogen and Protein Analyzer, Interscience, Breda, The Netherlands), using a nitrogen-to-protein conversion factor of 6.25 [22]. The use of the universal nitrogen-to-protein conversion factor of 6.25 was considered an estimate for the mixed meat–pulse matrix, given that nitrogen-to-protein conversion factors may vary depending on the protein source. Total fat content was determined according to the Soxhlet method. The solvent used was petroleum ether, and the extraction was performed in a Soxhlet extractor (Tecator Soxtec System HT 1043 Extraction unit plus Tecator Soxtec System HT 1046 Service unit, Hoganas, Sweden). Crude fat was determined gravimetrically after solvent evaporation in a rotary evaporator and drying in an oven [27].

Moisture content was determined at 105 °C (Binder, ED056, Tuttlingen, Germany) until a constant weight was obtained [28]. Total ash content was determined by incineration at 550 °C in a muffle furnace, until the ashes turned white [29]. Carbohydrate content was estimated by difference from the mean values of moisture, crude protein, fat and ash contents.

Mineral content was determined by inductively coupled plasma optical emission spectrometry (ICP-OES), using a Thermo Scientific iCAP 7000 Series instrument (Thermo Fisher Scientific, Waltham, MA, USA) [30]. Approximately 0.5 g of sample was digested with 8 mL of hydrochloric acid (37%) and 2 mL of nitric acid (65%) overnight, adjusted to 50 mL with deionized water and analyzed by ICP-OES. The same procedure was applied to the collected supernatant obtained after the in vitro digestion process (Section 2.6) to determine mineral in vitro bioaccessibility.

The percentage of mineral in vitro bioaccessibility was calculated by comparing the concentration of each mineral measured in the supernatant after in vitro digestion with its corresponding total concentration in the undigested sample and expressing this value as a percentage.

### 2.6. In Vitro Digestibility

In vitro digestibility was assessed following the INFOGEST protocol [31], with modifications. Samples were mixed with simulated salivary fluid (SSF) at a 1:1 (*w*/*w*) ratio containing α-amylase (75 U/mL) and incubated for 2 min at pH 7. Subsequently, simulated gastric fluid (SGF) containing pepsin (2000 U/mL) was added, and the mixture was incubated for 2 h at pH 3. The gastric digest was then combined with simulated intestinal fluid (SIF) supplemented with pancreatin (100 U/mL) and bile salts (10 mM) and incubated for an additional 2 h at pH 7.

All digestion steps were carried out at 37 °C under constant agitation using a rotating wheel. At the end of the intestinal phase, digestion was stopped by placing the samples on ice. The reagent blank contained the simulated digestive fluids and enzymes without sample and was processed in parallel to correct the measured mass and protein values. The digested samples were centrifuged for 20 min at 18,000× *g* and 4 °C, and the undigested residues were collected and dried overnight at 60 °C. All analyses were performed in triplicate, using three technical replicates from a single production batch for each formulation. Results were expressed on a dry-matter basis. Total in vitro digestibility (TIVD) was calculated according to Equation (3):

(3) $$TIVD\;\left(\%\right)=\;\left[\left(initial\;sample\;mass-residue\;mass\;corrected\;for\;the\;blank\right)/initial\;sample\;mass\right]\;\times 100$$

In vitro protein digestibility (IVPD) was calculated according to Equation (4). Crude protein content was determined from total nitrogen using the Dumas combustion method, with a nitrogen-to-protein conversion factor of 6.25.

(4) $$IVPD\;\left(\%\right)=\left[\left(initial\;crude\;protein-residual\;protein\;corrected\;for\;the\;blank\right)/initial\;crude\;protein\right]\times 100$$

### 2.7. Folin–Ciocalteu-Reactive Material and Antioxidant Activity Determination

Extracts for the Folin–Ciocalteu-reactive material and antioxidant assays were prepared using methanol–water solution (80:20, *v*/*v*), where 10 mL was added to 2 g of sample, and the mixture was shaken for 2 h. The supernatant was separated by centrifugation at 18,000× *g* at 20 °C for 10 min, and a new extraction was performed twice. The supernatant was stored at 4 °C until use. All the analyses were performed in the microplate reader CLARIOstar^®^ *Plus* (BMG LABTECH, Ortenberg, Germany).

Folin–Ciocalteu-reactive material was determined according to the method described by Al-Duais et al. [32] with some modifications, using the Folin–Ciocalteu reagent. In a microplate, 100 μL of Folin reagent was added to 20 μL of the samples. After 5 min, 80 μL of ${Na}_{2}CO_{3}$ was added, and the absorbance was read at 760 nm after 2 h of incubation in the dark. The Folin–Ciocalteu-reactive material was expressed in gallic acid equivalents per g using a calibration curve of gallic acid.

The supernatant was also used for the antioxidant analysis using the DPPH and FRAP methods. For both, a calibration curve was previously done using Trolox, and the results were expressed in Trolox equivalents per g. Antioxidant activity analysis by the FRAP method is based on the Carrasco-Sandoval et al. [33] method with some modifications. FRAP reagent is produced by mixing 0.5 mL of TPTZ (ferric 2,4,6-tripyridyl-s-triazine), 0.5 mL of FeCl_3_ and 5 mL of acetate buffer, then left for 15 min at 37 °C. In a microplate, 175 μL of the FRAP reagent was added to 25 μL of sample and then placed in the dark for 30 min before measuring the absorbance at 595 nm.

Antioxidant activity analysis by DPPH was based on the method described by Herald et al. [34] with some modifications. A total of 20 μL of the sample was added to 180 μL of DPPH solution (150 μmol L^−1^) in methanol–water (80:20, *v*/*v*) for 60 s in a 96-well microplate. After 40 min in the dark at room temperature, the absorbance was measured at 515 nm. All analyses were performed in triplicate, using three technical replicates from a single production batch for each formulation.

### 2.8. Nitrogen Footprint Calculation

The nitrogen (N) footprint of each formulation was estimated using the virtual nitrogen factor (VNF) approach of the N-Print model [18] and the CAPRI (Common Agricultural Policy Regionalised Impact) model [35]. The functional unit was 100 g of finished product, and calculations were based on the exact recipe of each formulation (76% pork meat for the control; 50% meat replacement by a chickpea flour/red lentil flour/potato protein isolate blend for both hybrid formulations, as described in Section 2.1).

The nitrogen footprint of each formulation was calculated according to Equation (5).

(5) $$NFP=\sum \left(m_{i}\times N_{i}\times {VNF}_{i}\right)$$

where $m_{i}$ is the ingredient quantity (g/100 g finished product), $N_{i}$ its nitrogen content (g N/100 g ingredient, derived from crude protein content using the nitrogen-to-protein conversion factor of 6.25, consistent with Section 2.5), and ${VNF}_{i}$ its ingredient-specific virtual nitrogen factor, defined as the ratio of reactive nitrogen lost during agricultural production to nitrogen retained in the edible product.

Protein values were obtained from the supplier technical data sheets (Table 1) or from USDA values [36]. The reference protein contents were 18.95 g/100 g for pork meat, 22.39 g/100 g for chickpea flour and 24.6 g/100 g for red lentils, corresponding to approximately 3.03, 3.58 and 3.94 g N/100 g, respectively. VNF values were obtained from Leach et al. [18]: 4.7 g N lost/g N in product for pork meat, 0.7 for pulses (chickpea and lentil) and 1.5 for potato-derived ingredients. These values represent average EU/US values and were applied irrespective of the country of origin of each ingredient batch. Minor nitrogen-free ingredients, including water, salt, oil, psyllium, carrageenan, colourant and aroma, were considered to make a negligible contribution to the nitrogen footprint. Sodium tripolyphosphate, its natural source substitute, and nitrate were likewise considered minor contributors.

The system boundary followed the farm-gate/soil-surface nitrogen budget approach of CAPRI [35], covering nitrogen inputs (mineral fertilizer, manure, biological fixation, atmospheric deposition) and nitrogen removed in harvested crops, but excluding downstream processing, packaging, transport and retail losses. No allocation was made between co-products of the same crop (e.g., protein isolate versus starch fraction); VNF values represent an average across the whole commodity. Trade and transport of imported ingredients were not modelled separately.

As VNF and nitrogen investment factors vary across production systems, regions, and years [18,35], the reported values are modelled estimates, not direct measurements, and should be interpreted as representative rather than site-specific. A formal uncertainty or sensitivity analysis was not performed and is recommended for future work.

### 2.9. Sensory Evaluation of the Hams

A sensory evaluation of control, hybrid and clean-label hybrid hams was conducted using an untrained panel of 40 participants (22–76 years; 28 females and 12 males). The evaluation was conducted in a single session with individuals who were present at the Ferreira Lapa Building of the Instituto Superior de Agronomia (ISA), Portugal, on the day of the sensory evaluation and voluntarily agreed to participate. Evaluations were carried out in individual sensory booths in accordance with ISO 8589:2007 [37]. All participants provided informed consent and the study was approved by the Ethics Committee of the Instituto Superior de Agronomia, Portugal (Opinion No. 3/2025). As part of the informed consent form, participants declared that they had no known allergies.

The samples were kept under refrigeration (approximately 4 °C) until the sensory evaluation. Each sample was prepared as a triangular portion measuring approximately 7 cm^2^ and identified using a three-digit code. The samples were presented in a randomized order. Water was provided for palate cleansing between samples.

Participants evaluated appearance, texture, aroma, flavour, and overall impression using a five-point hedonic scale ranging from “I liked it very much” (5) to “I disliked it very much” (1). Participants also rated their purchase intention on a five-point scale, ranging from “I would definitely buy” (5) to “I would definitely not buy” (1).

### 2.10. Statistical Analysis

All data, with the exception of the sensory evaluation results, were analyzed by analysis of variance (ANOVA), followed by Tukey’s test, with a 5% significance level (*p* < 0.05). As a single production batch was manufactured for each formulation (Section 2.1), the reported replicates correspond to technical replicates within each batch rather than independent replicates. Consequently, these statistical comparisons reflect measurement variability within a single batch of each formulation and should be interpreted as indicative of differences between formulations under the specific production conditions tested, rather than as generalizable inferences across independent production runs. Confirmation using independent production batches will be required to assess reproducibility across manufacturing runs in future studies. Sensory evaluation data were analyzed using the Friedman test, followed by Dunn’s multiple-comparison test. All statistical analyses were performed using GraphPad Prism, version 5 (GraphPad Software, San Diego, CA, USA).

## 3. Results

**Figure 1:**
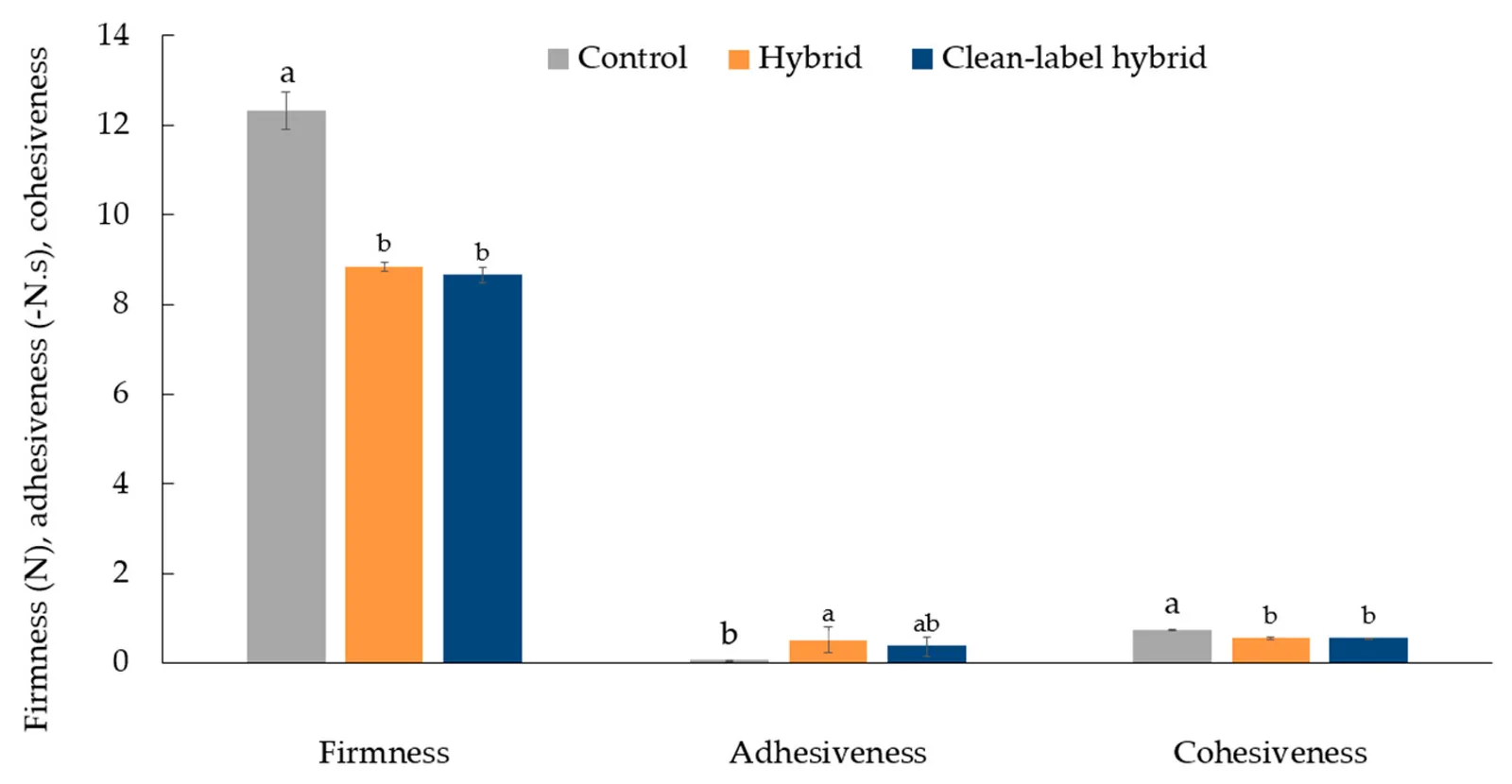
Firmness, adhesiveness and cohesiveness for control, hybrid and clean-label hybrid hams obtained by a Texture Profile Analysis. Data were obtained from five technical replicates from a single production batch per formulation. Different letters indicate differences between samples (*p* < 0.05, Tukey’s test) for each parameter.

Figure 1 presents the Texture Profile Analysis results for the control, hybrid, and clean-label hybrid ham samples. The control sample presented 28% higher firmness values (*p* < 0.05) than the hybrid formulations, in which 50% of the pork meat mass was replaced by a mixture of plant proteins. A similar pattern was observed for cohesiveness, with the control showing values approximately 25% higher than those of the hybrid formulations. A similar decreasing trend in cohesiveness and firmness was observed by Kamani et al. [38] in sausage formulations when meat was completely replaced by a mixture of plant-based proteins. Similarly, Chandler and McSweeney [39] verified that increasing the addition of pulse flours to hybrid chicken meat burgers decreased firmness and cohesiveness.

The lower firmness and cohesiveness observed in the hybrid formulations may be associated with differences in the composition and structure of the product matrix resulting from the partial replacement of meat by plant-based ingredients. These differences may have contributed to a matrix with greater compliance and greater capacity to accommodate deformation. This is also consistent with the findings of Argel et al. [11] who reported that the combination of higher pulse flour and higher water additions resulted in less firm burgers. For adhesiveness, the control sample showed similar values to the clean-label hybrid ham, suggesting that both formulations required a similar amount of work to overcome the attractive forces between the surface of the ham and other materials with which it comes into contact.

The hybrid and clean-label hybrid formulations did not differ from each other for any of the evaluated texture parameters. However, given the multicomponent nature of the formulations, these results should not be interpreted as evidence that the removal of carrageenan or the replacement of sodium tripolyphosphate alone had no effect on texture. Rather, the overall similarity in texture between the two hybrid formulations indicates that their combined formulation differences resulted in a comparable texture under the conditions evaluated.

A few results have been reported regarding the texture of meat products with added plant-based proteins, which varies according to the added protein, concentration, composition and intrinsic characteristics [40]. According to Youssef and Barbut [41], non-meat proteins can act by reducing firmness, due to their function as a binding agent for water and fat. However, Janardhanan et al. [42] verified that the decrease in texture attributes may be due to the weaker interaction between myofibrillar and plant-based proteins when analyzing meat-based, plant-based and hybrid patties.

**Figure 2:**
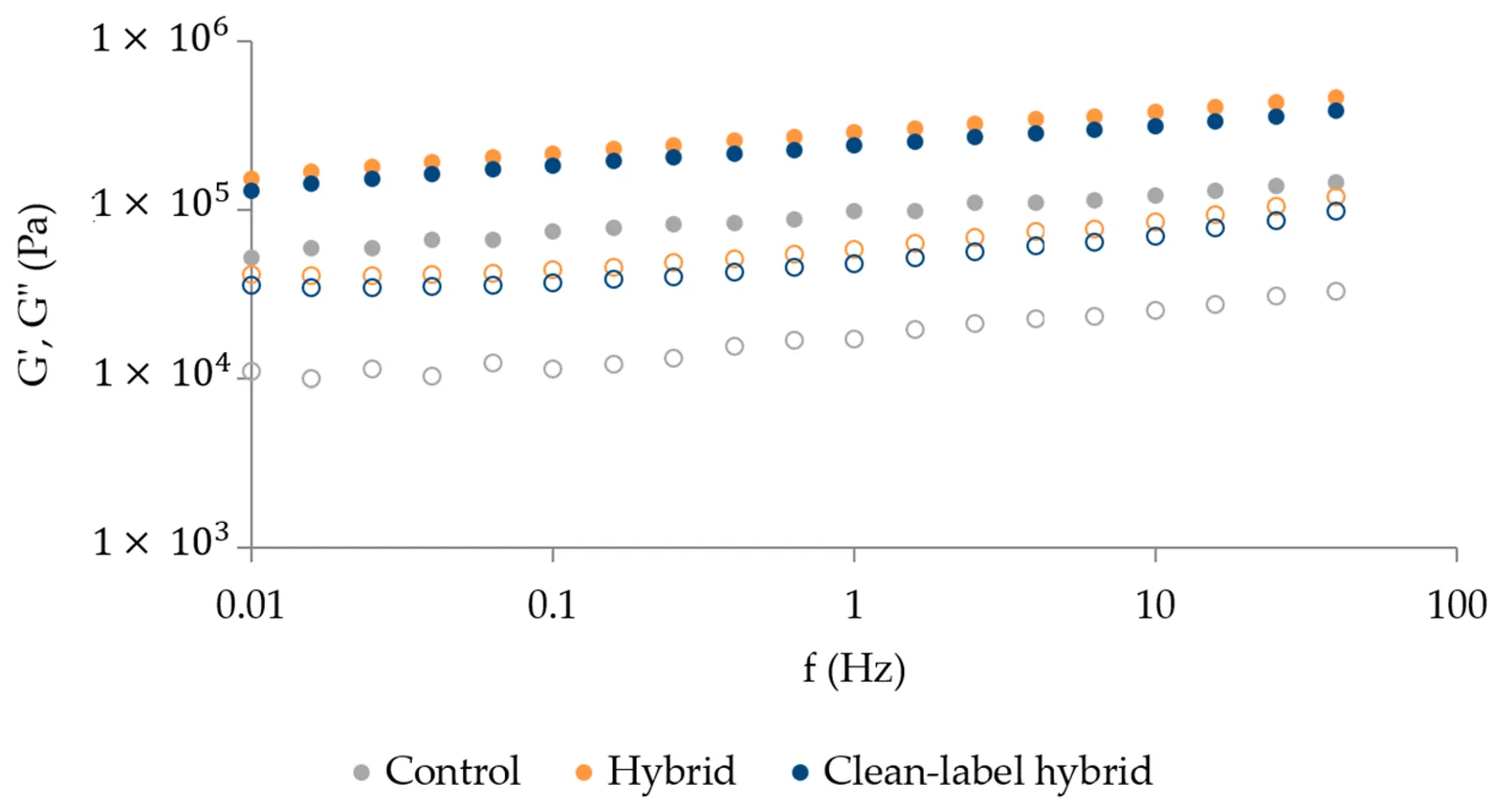
Mechanical spectra at 20 °C of control, hybrid and clean-label hybrid hams. G′ (full) corresponds to the storage modulus and G″ (open) corresponds to the loss modulus.

The evaluation of material behaviour through viscoelastic properties by mechanical spectra, obtained by small-amplitude oscillatory shear (SAOS), provides important information about the structural stability and gel network of processed meat products. The mechanical spectra of the different control, hybrid and clean-label hybrid ham samples are represented in Figure 2.

As shown in Figure 2, all samples exhibited similar mechanical spectra, with the storage modulus (G′) higher than the loss modulus (G″), indicating a predominantly elastic behaviour typical of structured meat systems [43,44]. The rheological characterization of the control and clean-label hybrid formulations was previously reported in [17]. These results are included here to provide the technological characterization of the three formulations within the broader scope of the present study. For comparison purposes, G′ values at 1 Hz were considered. Although the control formulation exhibited greater variability in G′ values at 1 Hz (81,056.63 ± 24,440.04 Pa) compared with the hybrid (286,709.78 ± 39,999.14 Pa) and clean-label hybrid (234,872.53 ± 23,389.26 Pa) samples, its mean G′ value remained lower than that of the hybrid formulations. No differences were observed between the hybrid and clean-label hybrid formulations under the conditions evaluated. Given the multicomponent nature of the formulations, the comparable G′ values indicate that the overall differences between the two hybrid formulations resulted in similar elastic behaviour under the conditions evaluated. These findings are consistent with previous studies reporting that natural ingredients can partially replace conventional additives while maintaining comparable rheological properties [45,46].

The higher G′ values observed for the hybrid formulations indicate a higher elastic modulus under the small amplitude deformation conditions applied during the rheological analysis. In contrast, the texture profile analysis showed that the control formulation was approximately 28% firmer than the hybrid formulations. This apparent difference can be explained by the distinct deformation regimes of the two techniques. Oscillatory rheology characterizes the viscoelastic response of the samples under small amplitude deformation within the linear viscoelastic region, whereas TPA involves substantially larger deformation and may reflect local structural failure, fracture and resistance to penetration.

The higher elastic modulus under the small amplitude deformation for the hybrid formulations may be associated with the contribution of vegetable proteins to the development of the composite gel network. Badia-Olmos et al. [47] reported that chickpea flour and red lentils can develop structured systems with predominantly solid-like behaviour. Pork meat proteins, particularly myofibrillar proteins such as myosin, also have the ability to form gel networks upon heating. When combined, these components may generate additional protein–protein interactions and increase the elastic response of the matrix under small deformation.

These results suggest that the incorporation of vegetable proteins may have modified the organization and mechanical response of the meat-based gel network, particularly under small deformation. Indeed, when vegetable proteins are heated, protein molecules undergo denaturation and structural rearrangement, which can facilitate intermolecular interactions and gel formation during cooling [47,48]. The interactions between vegetable proteins and meat proteins could therefore contribute to the development of a more interconnected composite gel network, which may explain the higher G′ values observed. This is in line with Farooq and Boye [12], who suggested that pulse proteins and starch may absorb water from gel matrices upon heating and interact with other food matrix components, such as meat proteins, potentially forming a complex three-dimensional gel network in which starch and non-meat proteins act as fillers.

**Figure 3:**
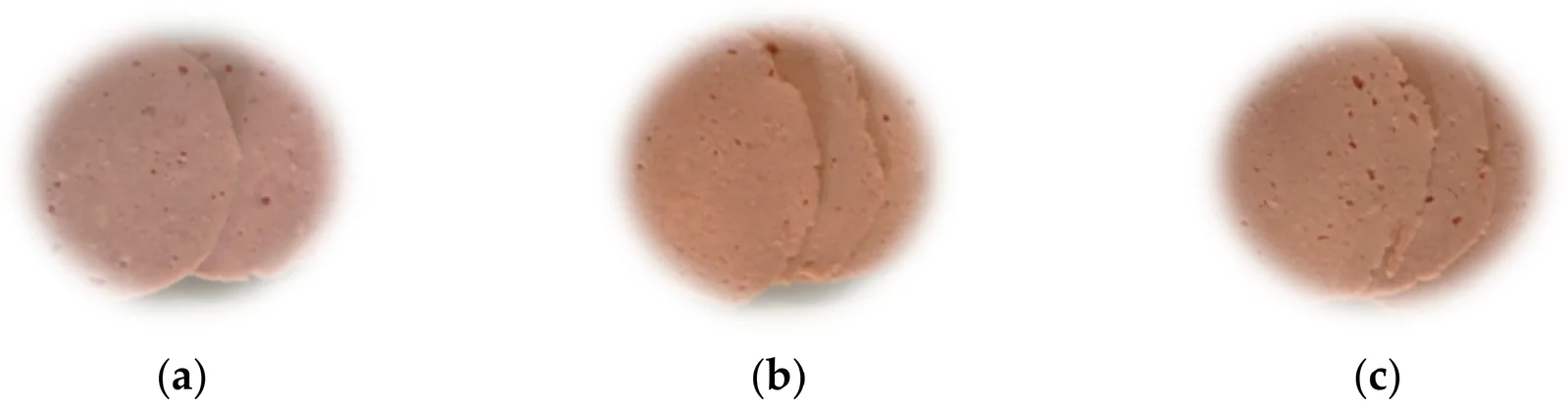
Images of control (**a**), hybrid (**b**) and clean-label hybrid (**c**) hams.

For the colour testing, according to the results in Table 3 and Figure 3 it is possible to verify that the control and hybrid hams showed differences (*p* < 0.05) in colour parameters, with the control ham having a lighter appearance and a less intense red colour. To evaluate colour differences, using the ΔE value, the calculation gives a value greater than 5, meaning that the colour difference is perceptible by the human eye [25].

Cooked ham has a light pink colour due to the addition of nitrate; during cooking the colour of the ham changes from red (pork meat) to pink. This change in colour depends mainly on the myoglobin content present, which depends on the type of muscle and the age of the animal [49].

Nitrate was maintained in the hybrid ham formulation, playing a less intense role in this ham, since the meat was replaced by 50%. In this case, it was observed that the colour of the pulse flours was what determined the colour of the ham. The addition of red lentil flour contributes not only to the nutritional characteristics, but also acts as a natural colouring agent.

**Figure 4:**
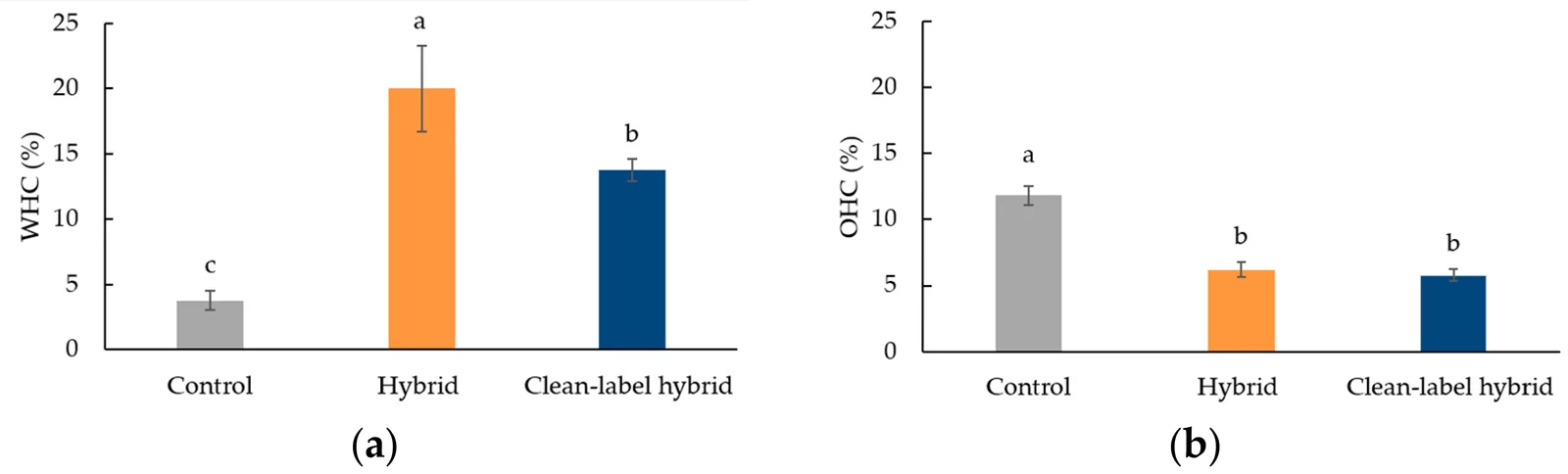
Water holding capacity (WHC) (**a**) and oil holding capacity (OHC) (**b**) for the control (grey), hybrid (orange) and clean-label hybrid (blue) samples. Data were obtained from three technical replicates from a single production batch per formulation. Samples marked with different letters showed different values (*p* < 0.05, Tukey’s test) for each parameter.

Water holding capacity (WHC) and oil holding capacity (OHC) are important technical functional characteristics of the samples and are shown in Figure 4. WHC has an influence on the sensory traits during consumption, visual acceptability of the products, texture, juiciness and flavour [50]. The WHC is correlated with the amount of charged and polar amino acids since the charged amino acid side chains have a strong potential to bind water molecules [51].

Hybrid hams showed a higher WHC, which may be associated with the water-absorption capacity of the pulse flours; however OHC in the control sample was higher than for the hybrid hams. Also, according to Ma et al. [52] the OHC of the plant-based protein isolates was lower than the water holding capacity. This was expected because the proteins used have more hydrophilic amino acids than the myosin meat proteins, with a major share of charged and polar amino acid residues. The OHC influences the texture and sensorial properties, since fat acts as a flavour retainer and increases the mouthfeel of food [53]. According to Benítez et al. [54] the binding of oils depends on the surface availability of hydrophobic amino acids and other non-polar side chains.

As shown in Table 4, the clean-label hybrid ham presented a crude protein content of 18%, which was higher than that of both the hybrid ham and the control sample, which showed crude protein contents of approximately 14%. The higher crude protein content observed in the clean-label hybrid ham may be attributed to differences in the protein contribution of the ingredients used in its formulation, particularly the sodium tripolyphosphate substitute (51%).

The incorporation of plant protein ingredients such as chickpea and lentil flours has been widely reported as an effective strategy to improve the nutritional profile of hybrid meat products, as these pulses typically contain 13–30% protein and generally provide a relatively favourable amino acid profile. However, their proteins may be relatively limited in sulfur-containing amino acids, particularly methionine and cysteine [11,55,56]. Additionally, potato protein isolate is recognized for its high biological value and functional properties, including emulsification and water binding capacity, which can positively influence protein retention in processed meat systems [57,58,59,60].

Interestingly, Chandler and McSweeney [39] verified that the chickpea and lentil’s flours addition did not significantly decrease the protein content of hybrid meat burgers. This finding suggests that pulse incorporation can partially compensate for meat reduction, depending on inclusion levels and formulation design. Moreover, several studies have demonstrated that hybrid meat products containing pulse proteins can maintain comparable or even enhanced protein levels relative to traditional formulations [61,62].

Wi et al. [63] demonstrated that fat is important in hams due to its ability to retain flavour and enhance mouthfeel, juiciness and tenderness, attributes that influence consumer acceptance of hybrid products. In this study, since oil was incorporated into both hybrid samples, their lipid content (around 6%) was higher than that of the control (1.8%). Carvalho et al. [64], in the development of hybrid hams incorporating insect flour, reported higher lipid levels, approximately 40%. Similarly, Jankauskienė et al. [65] observed lipid content ranging from 24% to 29% in sausages enriched with insects, thereby highlighting the importance of lipid content in the formulation of hybrid products.

When comparing the moisture content, the hybrid samples (around 48%) showed lower moisture than the control (78%). These results are due to the fact that pork meat is higher in water content (74%) than the pulse flours (<15%) (Table 1). In fact, these pulse flours can absorb water molecules by bonding to the hydrophilic components of the starches and fibre; therefore there is a tendency to decrease the moisture content as the quantity of pulse flour increases [39,66].

Similarly, the pH and water activity (a_W_) values differed among the formulations, with the control, hybrid, and clean-label hybrid samples presenting values of 6.30 ± 0.03, 6.28 ± 0.02, and 6.50 ± 0.01, respectively, for pH. Regarding water activity, the control sample presented the highest value (0.97 ± 0.00), followed by the clean-label hybrid (0.96 ± 0.00) and hybrid (0.95 ± 0.00). The values of pH and a_W_ alone cannot be used to establish microbiological stability or product safety. However, these results provide a relevant physicochemical characterization of the formulations.

The a_W_ results showed an inverse trend relative to water holding capacity (Figure 4), which may be attributed to the stronger binding of water within the product matrix, thereby reducing the fraction of free water available to contribute to water activity. This is consistent with the rheological results, where the higher storage modulus (G′) observed in the hybrid samples indicated a more structured gel network. In this study, psyllium and pulse flours likely play a central role in this behaviour, as both ingredients present a high water holding capacity, retaining water more strongly and consequently decreasing the amount of free water available in the hybrid products. According to Fradinho et al. [67] a reduction in a_W_ values occurred with the increase in psyllium content in biscuits. Similarly, Kristensen and Jensen [68] reported that each gram of psyllium can retain approximately 10 g of water.

Regarding ash content, the hybrid sample showed the higher value, followed by the control sample thus reflecting the presence of phosphates and nitrates, as the clean-label hybrid ham did not have the addition of these compounds.

The hybrid samples showed a higher content of carbohydrates than the control. These results may be largely expected because the pulse flours used have carbohydrate contents of 48% and 56% (Table 1) due to the presence of starch and other polysaccharides. According to Li et al. [69] pulses are a rich source of nutrients that are important for human health, including complex carbohydrates (e.g., resistant starch and other dietary fibres), proteins, minerals and phytochemicals (e.g., isoflavones, phytosterols and saponins).

**Figure 5:**
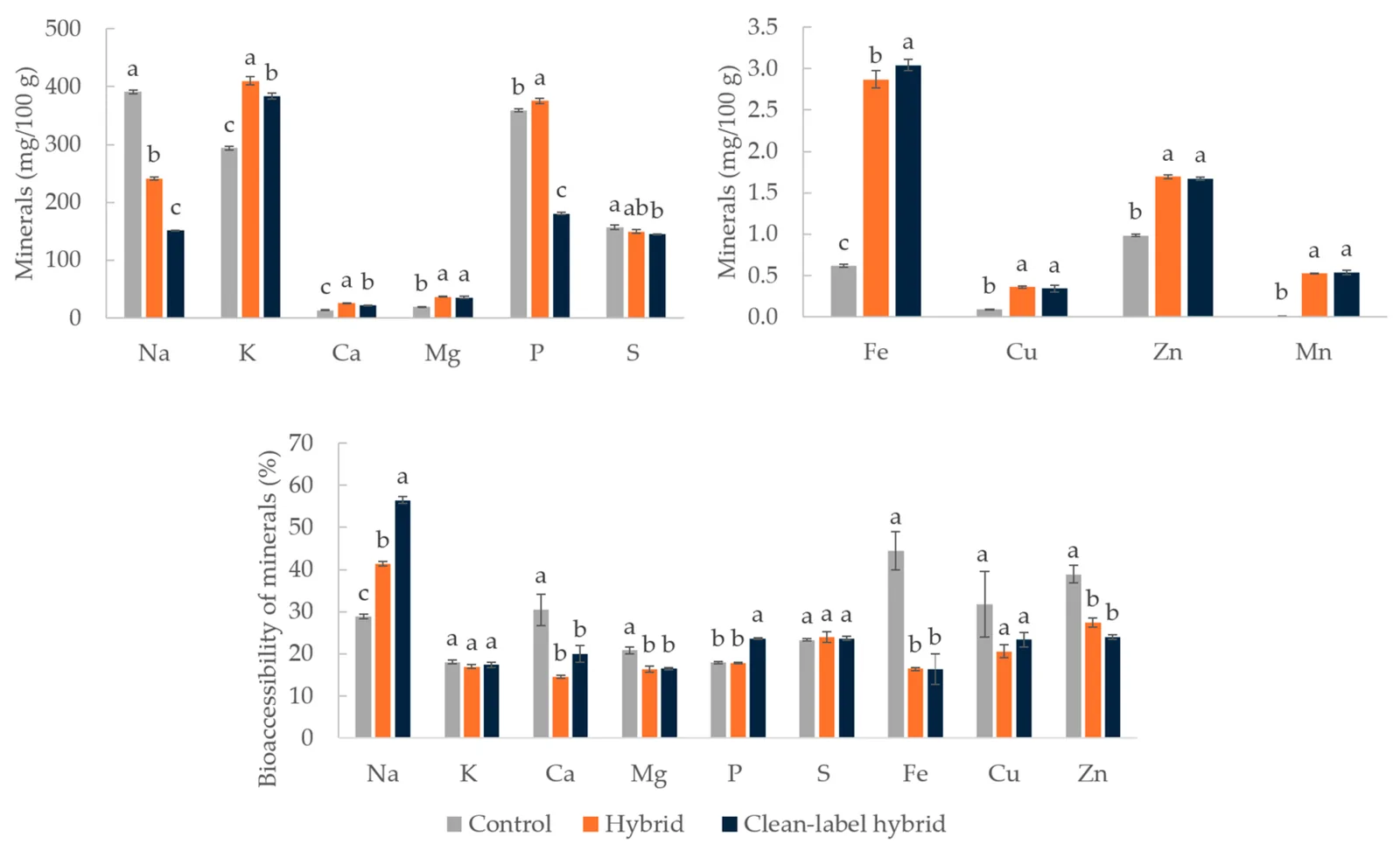
Mineral content in the undigested control, hybrid and clean-label hybrid hams and the corresponding in vitro bioaccessibility. Data were obtained from three technical replicates from a single production batch per formulation. Results marked with different letters showed different values (*p* < 0.05, Tukey’s test) for each parameter.

Figure 5 presents the mineral content of the samples prior to in vitro digestion, together with the corresponding in vitro bioaccessibility results, calculated relative to this initial content. Mineral in vitro bioaccessibility varied among the control, hybrid and clean-label hybrid formulations, highlighting the impact of product composition on nutrient availability. In general, minerals such as sodium, calcium, phosphorus, and zinc showed differences between formulations. In contrast, potassium, sulfur, and copper exhibited a more homogeneous behaviour, which suggests a lower influence of partial ingredient substitution on their availability.

For sodium, a progressive increase in in vitro bioaccessibility was observed from the control to the hybrids and, especially, to the clean-label hybrid formulation, indicating that modifications in the food matrix may favour mineral release during digestion. Calcium displayed higher bioaccessibility in the control sample (30.5%), with a reduction in the hybrid formulations (14.5–20%).

A similar trend was observed for magnesium, although with less pronounced differences (20.8% for the control and around 16% for the hybrids). These results, although lower, are consistent with those reported by Luz et al. [70], who found higher bioaccessibility of calcium (92.4%) and magnesium (83.6%) in meat-based burgers than in plant-based burgers (31.5% and 81%, respectively).

For phosphorus, the clean-label formulation presented higher values (23.6%) than the other samples (around 18%), indicating a difference in phosphorus in vitro bioaccessibility among the formulations under the conditions tested. Among the trace minerals, iron showed the highest in vitro bioaccessibility in the control formulation (44.5%), with a reduction in the hybrid products (around 16%). This difference may be related, at least in part, to the presence of heme iron in meat, which is generally more readily absorbed than non-heme iron, as well as to the presence of potential mineral-binding compounds in plant ingredients, such as phytate, oxalate and condensed tannins, which may reduce mineral release during digestion, as reported in the literature [71,72].

A similar pattern was observed for zinc, whose highest value occurred in the control sample (39%), which may reflect a greater capacity of the meat matrix to provide this essential mineral under the conditions tested. Nevertheless, the maintenance of moderate levels in hybrid formulations (24–27%) may reflect differences in the composition and structure of the formulations resulting from interactions between animal and plant proteins [73].

Under the conditions tested, the results confirm that meat products tend to present higher in vitro bioaccessibility of iron and zinc, whereas hybrid formulations may positively modulate the availability of other minerals, depending on the plant composition used. Although plant-based ingredients may contain antinutritional compounds capable of reducing mineral absorption, certain matrices demonstrate good in vitro bioaccessibility. For example, chickpea-based plant burgers described in the literature show in vitro bioaccessibility values ranging from 31.5% to 81.0% for Ca, Mg, Mn and Zn [70,72]. In this context, hybrid products represent a promising strategy to balance nutritional quality with reduced meat content, and further studies are needed to optimize formulations and maximize mineral in vitro bioaccessibility.

**Figure 6:**
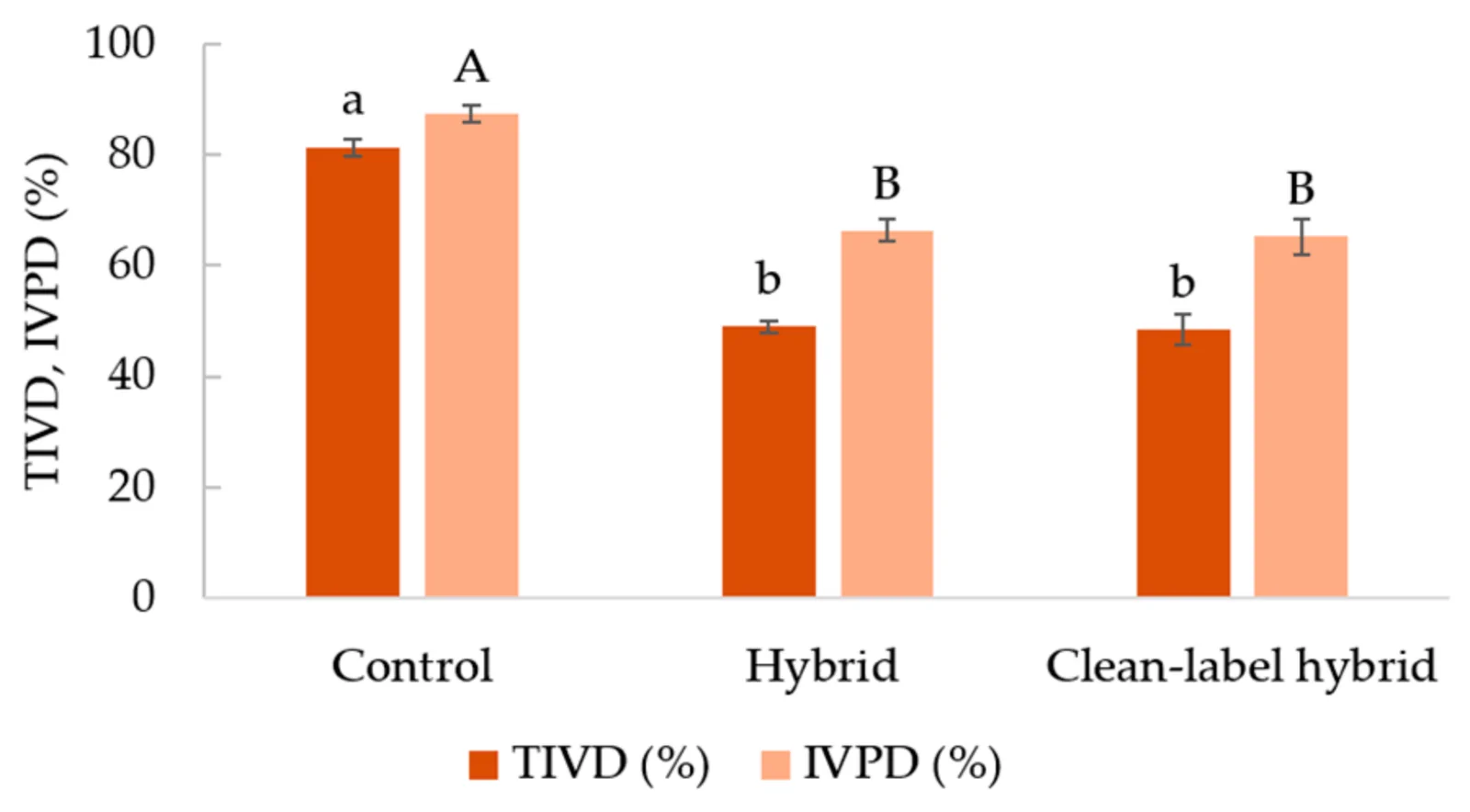
Total in vitro digestibility (TIVD) and in vitro protein digestibility (IVPD) of control, hybrid and clean-label hybrid hams. Data were obtained from three technical replicates from a single production batch per formulation. Different lowercase letters indicate differences in TIVD and uppercase letters indicate differences in IVPD (*p* < 0.05), according to Tukey’s test.

Figure 6 shows that the control ham presented higher values of total in vitro digestibility (TIVD) and in vitro protein digestibility (IVPD) (81.4% and 87.4%, respectively) compared to the hybrid hams (around 48% and 66%, respectively), representing a substantial reduction of approximately 33% and 21%, respectively. This finding was expected, as according to Xie et al. [74] and Sarwar Gilani et al. [75], animal-derived proteins generally exhibit higher digestibility than plant proteins due to their balanced essential amino acid profile, lower content of antinutritional factors, and greater susceptibility to gastrointestinal enzymatic hydrolysis. Similarly, Schaafsma [76] reported digestibility values frequently exceeding 90% for animal proteins, whereas several plant protein sources present lower digestibility depending on origin and processing conditions.

Plant proteins may show reduced digestibility because of the presence of compounds such as phytates, tannins and protease inhibitors, which can limit the activity of digestive enzymes, including pepsin and trypsin [75]. Therefore, the reduced TIVD and IVPD observed in the hybrid hams may be associated with the structural complexity of plant proteins, which, according to Opazo-Navarrete et al. [77] and Cutroneo et al. [78] may be embedded within cell wall structures or associated with other constituents, such as carbohydrates, that act as physical barriers to enzymatic action, thereby reducing the fraction of protein hydrolyzed during in vitro digestion.

Moreover, interactions between plant and animal proteins may modify the product microstructure and further influence overall digestibility. Baugreet et al. [79] studied the digestibility of hybrid meat products composed of a pea protein isolate combined with rice and beef proteins and found that plant-based proteins altered the microstructure of the hybrid products compared to the 100% meat control, thereby affecting protein digestibility by reducing peptide release during digestion.

Nevertheless, the digestibility levels observed in the hybrid formulations remain nutritionally relevant, supporting their technological feasibility and potential as partially meat-reduced products. Further research is still required to fully understand the interactions between different protein sources and their impact on digestibility.

Folin–Ciocalteu-reactive material values, shown in Table 5, are higher in the hybrid (0.5 mg GAE/g) and clean-label hybrid (0.4 mg GAE/g) samples, which may be related to the presence of pulse flours, such as chickpea and red lentils, which contain phenolic compounds. Ladjal and Chibane [51] verified the phenolic content of different pulse flours, and the lentil extract showed the highest reported value of 6.21 mg GAE/g followed by pea (2.36 mg GAE/g) and chickpea (1.50 mg GAE/g). Phenolic compounds are plant-derived constituents that may contribute to chemical antioxidant activity [80,81].

It is important to note that the control formulation also showed a Folin–Ciocalteu response when analyzed using the Folin–Ciocalteu (F–C) method. Although these are not typically expected to be present in meat, F-C interferents such as tyrosine and other reducing compounds, including organic acids, may be present [82,83].

With respect to antioxidant activity, the hybrid samples demonstrated higher values compared to the control. These results indicate greater chemical radical-scavenging and reducing capacity of the hybrid formulations under the conditions of the respective assays [84,85].

According to the exact recipe of each of the ham formulations, the estimated N footprints were: 10.8 g N/100 g for the control ham produced with meat, 6.76 g N/100 g for the hybrid formulation, and 9.7 g N/100 g for the clean-label hybrid. Under the assumptions and system boundaries applied in the present model, the hybrid formulations showed lower estimated nitrogen footprint values than the control. This difference reflects the formulation-specific nitrogen factors assigned to the animal- and plant-derived ingredients and should therefore be interpreted as a modelled difference rather than as a direct measurement of nitrogen losses. The partial substitution of meat with plant-based protein sources was associated with a lower estimated nitrogen footprint in the model, rather than demonstrating improved nitrogen-use efficiency [86,87].

Although both hybrid formulations contained the same proportion of meat replacement (50% of pork meat mass), the clean-label hybrid exhibited a higher estimated nitrogen footprint than the hybrid. This result may initially appear unexpected, as clean-label products are often perceived as more environmentally sustainable.

However, in this case, the higher estimated nitrogen footprint in the clean-label hybrid may be related to the distinct ingredient composition and the nitrogen-conversion factors considered in the model. The results should thus be interpreted as model-based estimates specific to the ingredients and system boundaries considered in this study. Since the nitrogen footprint model attributes reactive nitrogen losses to agricultural production of protein-containing raw materials, a higher demand for such inputs leads to greater upstream nitrogen losses, which are reflected in the final product footprint [18,20,88]. As reported by Rautiainen et al. [89] and Pierer et al. [88] proteins are the main nitrogen-containing compounds in food systems; consequently, the nitrogen footprint is strongly influenced by the protein content of the raw materials, although it ultimately reflects the nitrogen losses associated with their agricultural production rather than protein content alone.

These findings demonstrate that nitrogen footprint estimates are sensitive to formulation composition and to the nitrogen factors assigned to individual ingredients. However, the present calculation represents only one environmental indicator and does not establish overall environmental sustainability. A broader environmental assessment would require additional indicators, including greenhouse-gas emissions, land use, water use, energy demand, and processing and packaging impacts.

**Figure 7:**
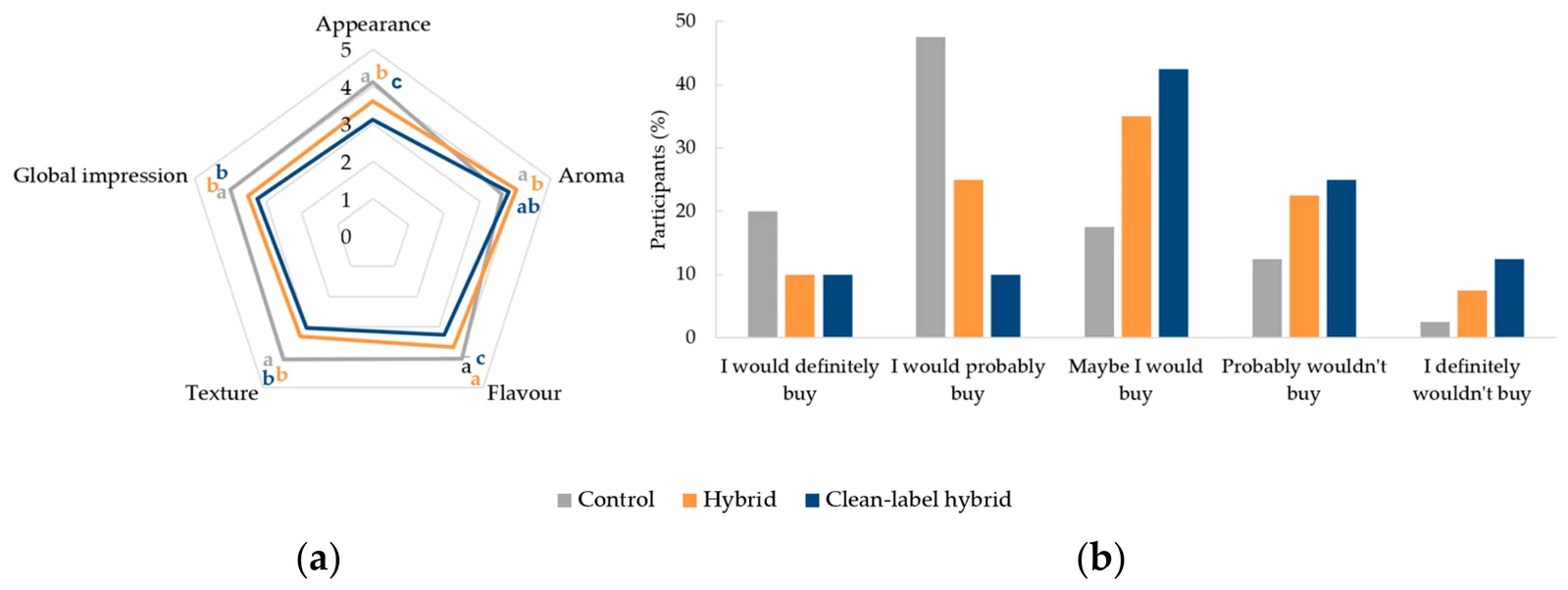
Sensory acceptance (**a**) and purchase intention (**b**) of control, hybrid and clean-label hybrid hams. Different letters indicate significant differences between results (*p* < 0.05) according to the Friedman test followed by Dunn’s multiple-comparisons test. The figure presents responses from 40 participants who evaluated all three formulations.

Figure 7 shows the sensory acceptance and purchase intention results for the control, hybrid, and clean-label hybrid hams. Overall, the sensory responses differed among formulations for several evaluated attributes, indicating that sensory acceptance was not uniform across formulations. For aroma, the clean-label hybrid formulation did not differ significantly from either the control or the hybrid formulation. In contrast, differences were observed for appearance, flavour, texture, global impression and purchase intention. Therefore, the sensory results suggest formulation-dependent changes rather than a uniformly maintained level of sensory acceptance across all attributes.

In the present study, hybrid hams were formulated with a 50% replacement of meat using chickpea flour, red lentil flour and isolated potato protein. This substitution level lies at the upper boundary of the moderate replacement range reported in the literature (20–50%), within which hybrid meat products may still retain favourable sensory attributes such as flavour, juiciness and texture. On the other hand, higher incorporation of plant-based ingredients is often associated with reduced consumer acceptance due to off-flavours, colour deviations and texture problems. Consistently, these ingredients can introduce characteristic beany or earthy notes when used at elevated levels, negatively affecting flavour perception. However, functional protein ingredients and appropriate structuring strategies may partially compensate for these effects by enhancing gel formation, firmness and water holding capacity, which are key determinants of texture and juiciness in hybrid meat systems [13].

Supporting these observations, Chin et al. [90] reported that hybrid burgers containing 50% meat retained favourable flavour and texture characteristics, although slight differences in elasticity were observed compared with conventional meat products. Conversely, when the plant-based content exceeded 50%, consumer acceptance declined due to off-flavours, suboptimal textures and colour inconsistencies. This trend underscores a broader consumer preference for sensory familiarity and highlights the importance of targeted product development strategies to optimize flavour, texture and visual appeal in hybrid products with higher plant content [91].

Consumer perception and acceptance play a pivotal role in the market success of hybrid meat products. Consumers evaluate these products across multiple dimensions, including flavour, texture, appearance and overall familiarity, reflecting the complexity of expectations in this emerging food category. Although motivations such as health, sustainability and novelty drive interest, concerns about flavour, unfamiliar ingredients, processing, and price can limit acceptance, underscoring the importance of consistent sensory appeal [92].

Within the clean-label context, studies have shown that reducing or replacing synthetic additives in meat products can be achieved without significant impairment of sensory or microbiological characteristics when appropriate technological strategies are applied [46]. Similarly, reformulated hybrid hams using clean-label ingredients have demonstrated comparable texture and colour sensory acceptability to conventional formulations. Moreover, the use of natural antimicrobial agents has been proposed as an effective approach to preserve microbiological stability in products with reduced or natural additives, highlighting the feasibility of reconciling product naturalness with quality and safety [93,94]. In the present study, microbiological safety and refrigerated shelf life were not evaluated. Although pH and water activity were determined as part of the physicochemical characterization, these parameters alone are not sufficient to establish microbiological stability or product safety. Therefore, the formulations should be considered experimental prototypes, and further studies under refrigerated storage conditions are required to assess their microbiological safety and determine their shelf life.

As can be seen in Figure 7b, the purchase intention responses differed among formulations. The proportion of participants selecting either “I would definitely buy” or “I would probably buy” was 68% for the control formulation, 35% for the hybrid formulation, and 20% for the clean-label hybrid formulation. In contrast, the proportion selecting “Maybe I would buy” increased from 18% for the control to 35% and 43% for the hybrid and clean-label hybrid formulations, respectively. These results indicate a progressive reduction in purchase intention as formulation modifications were introduced and the transition to a clean-label occurred. These values are aligned with previous research on consumer responses to hybrid meat products and suggest that purchase intention generally declines as plant substitution increases or as products diverge from familiar meat characteristics [95].

The present findings demonstrate the technological feasibility of developing hybrid and clean-label hybrid hams using the proposed ingredient combinations, while also showing that formulation changes negatively affected several sensory attributes and purchase intention. Sensory responses were not uniform across formulations, and the purchase intention results highlighted the need for further formulation optimization to increase consumers’ confidence and willingness to purchase the modified products. Future studies involving larger and more diverse consumer panels, together with market-oriented evaluations, are needed to assess consumer acceptance and market potential more broadly.

## 4. Conclusions

Under the experimental conditions evaluated, the proposed formulations could be processed into sliceable deli ham prototypes and exhibited measurable differences in physicochemical, structural, nutritional and sensory characteristics. The hybrid formulations exhibited lower moisture content, lower in vitro bioaccessibility of several minerals, darker colour and lower in vitro digestibility, but also showed greater water holding capacity and a higher small-strain elastic modulus compared with the control. From a nitrogen-footprint perspective, the hybrid formulations showed lower estimated nitrogen-footprint values than the control under the assumptions and system boundaries applied in the model. Broader environmental and sustainability claims would require the assessment of additional environmental indicators. The incorporation of pulse flours was further associated with higher Folin–Ciocalteu-reactive material and greater antioxidant activity in the DPPH and FRAP assays. These changes were accompanied by differences in sensory responses among formulations and, notably, by lower purchase intention, indicating trade-offs associated with meat replacement and clean-label reformulation.

Taken together, these technological, compositional and sensory trade-offs indicate that further development is needed before these prototypes can be considered market-ready. Parameters relevant to industrial and commercial feasibility, such as cooking yield, syneresis, storage stability and microbiological safety, were not assessed in this study and should be addressed in future research, together with further formulation refinement and evaluation across independent production batches, before broader nutritional or sustainability claims can be made.

## Figures and Tables

**Table 1:** Chemical composition of the pulse flours and meat used in the products, as taken from the technical sheets.

Ingredient	Fat (%)	Protein (%)	Carbohydrates (%)	Fibres (%)	Salt (%)	Moisture (%)
Pork meat	2.6	22.1	NF	NF	0.6	74.3
Potato protein isolate	0.1	93.2	<0.1	0.2	0.04	<7
Red lentils	1.8	23.0	56.1	6.6	0.04	<15
Chickpea	6.5	19.3	48.4	10.8	0.02	<15

NF = not informed.

**Table 2:** Quantitative composition of the three deli ham formulations (g/100 g).

Ingredients	Control	Hybrid	Clean-Label Hybrid
Pork meat	76.0	29.5	29.5
Water	16.7	11.6	11.6
Ice	4.8	11.6	11.6
Salt	1.4	1.0	1.0
Sodium tripolyphosphate	0.7	0.7	-
Carrageenan	0.3	0.3	-
Nitrate	0.1	0.1	-
Chickpea flour	-	6.8	6.8
Red lentil flour	-	20.5	20.5
Isolated potato protein	-	2.5	2.5
Sunflower oil	-	10.6	10.6
Sodium tripolyphosphate substitute	-	-	1.3
Psyllium	-	3.0	3.0
Colourant	-	0.8	0.8
Aroma	-	0.8	0.8

**Table 3:** Colour parameters of control, hybrid and clean-label hybrid hams.

Sample	L*	a*	b*	ΔE
Control	${71.4\pm 0.33}^{\;a}$	${9.5\pm 0.16}^{\;b}$	${6.7\pm 0.39}^{\;c}$	-
Hybrid	${54.3\pm 0.33}^{\;b}$	${10.9\pm 0.13}^{\;a}$	${19.2\pm 0.36}^{\;a}$	18.7
Clean-label hybrid	${51.1\pm 0.21}^{\;c}$	${10.4\pm 0.30}^{\;a}$	${16.5\pm 0.86}^{\;b}$	18.2

Data were obtained from three technical replicates from a single production batch per formulation. Different letters in column indicate different results according to Tukey’s test.

**Table 4:** Chemical composition of the of control, hybrid and clean-label hybrid hams.

Nutritional Information		Content per 100 g	
	Control	Hybrid	Clean-Label Hybrid
Lipids (g)	1.8 ± 0.26 ^a^	6.8 ± 0.13 ^b^	6.3 ± 0.18 ^c^
Crude protein (g)	14.3 ± 0.37 ^b^	14.6 ± 0.41 ^b^	18.0 ± 0.87 ^a^
Ash (g)	3.0 ± 0.03 ^b^	3.1 ± 0.02 ^a^	2.6 ± 0.03 ^c^
Carbohydrates (g)	3.1	27.5	25.1
Energy (kcal/kJ)	85.2/356.9	229.9/963.1	228.9/959.2

Values represent means ± SD of three technical replicates from a single production batch per formulation. Results marked with different letters in same row showed different values (*p* < 0.05, Tukey’s test) for each parameter.

**Table 5:** Folin–Ciocalteu-reactive material and antioxidant activity for control, hybrid and clean-label hybrid products.

Sample	F–C-Reactive Material (mgGAE/g)	Antioxidant Activity (mgTE/g) FRAP	Antioxidant Activity (mgTE/g) DPPH
Control	0.18 ± 0.004 ^c^	0.03 ± 0.002 ^c^	0.04 ± 0.014 ^b^
Hybrid	0.50 ± 0.015 ^a^	0.29 ± 0.020 ^a^	1.19 ± 0.089 ^a^
Clean-label hybrid	0.40 ± 0.050 ^b^	0.24 ± 0.012 ^b^	1.10 ± 0.223 ^a^

The results of DPPH and FRAP analysis are expressed in mg trolox equivalents (TE)/g and for Folin–Ciocalteu-reactive material analysis are expressed in mg gallic acid equivalents (GAE)/g. Values represent means ± SD of three technical replicates from a single production batch per formulation. Different letters in column indicate different results according to Tukey’s test.

## Data Availability

The original contributions presented in the study are included in the article; further inquiries can be directed to the corresponding author.
